# Strategy to enhance the therapeutic effect of doxorubicin in human hepatocellular carcinoma by selenocystine, a synergistic agent that regulates the ROS-mediated signaling

**DOI:** 10.18632/oncotarget.1854

**Published:** 2014-03-25

**Authors:** Cundong Fan, Wenjie Zheng, Xiaoyan Fu, Xiaoling Li, Yum-Shing Wong, Tianfeng Chen

**Affiliations:** ^1^ Department of Chemistry, Jinan University, Guangzhou 510632, China; ^2^ School of Life Sciences and State Key Laboratory of Agrobiotechnology, The Chinese University of Hong Kong, Hong Kong S.A.R., China

**Keywords:** selenocystine, synergism, doxorubicin, apoptosis, reactive oxygen species

## Abstract

Doxorubicin-based chemotherapy represents one of the most effective ways in combating human cancers. However, its clinical use is limited by severe side effects. Selenocystine (SeC) is a natural available selenoamino acid with novel anticancer efficacy. In this study, we used SeC to sensitize HepG2 human hepatocellular carcinoma (HCC) cells to DOX, and to achieve anticancer synergism in vitro and in vivo. Treatment with DOX dose-dependently reduced HepG2 cell viability through initiating cell apoptosis and strong G2/M phase cell cycle arrest. Mechanistic studies indicated that this sensitization of SeC to DOX was achieved by triggering inactivation of ERK and AKT and DNA damage through reactive oxygen species (ROS) overproduction. Pretreatment with inhibitors of ERK and AKT markedly enhanced combined treatment-induced cell killing, indicating that combined treatment-induced HCC cell killing with ERK- and AKT-dependent manner. Furthermore, inhibition of ROS effectively attenuated combined treatment-induced DNA damage and inactivation of ERK and AKT. Additionally, xenograft hepatocellular carcinoma growth was also effectively inhibited by combined treatment through induction of cell apoptosis in vivo. Taken together, our results suggest that the strategy to use SeC and DOX in combination could be a highly efficient way to achieve anticancer synergism against HCC.

## INTRODUCTION

Human hepatocellular carcinoma (HCC) is the fifth most frequent neoplasm worldwide and the third leading cause of cancer-related mortality [1-4]. To date, systemic chemotherapeutic treatment is ineffective against HCC cells, due to the resistance to apoptosis and severe side-effect to normal tissues [5]. Therefore, new therapeutic strategies are urgently needed. Doxorubicin (DOX), an anthracycline antibiotic, is one of the most effective and widely used chemotherapeutic agent for treatment of human various malignancies, including metastatic breast cancer, lymphomas and sarcomas, as well as other human neoplasms [6, 7]. Nevertheless, the most common adverse effects limiting the clinical use of DOX are cardiotoxicity and even heart failure that carry a poor prognosis and are frequently fatal [6-8]. Increasing studies proved that the proposed mechanisms of anti-malignancy effects of DOX ascribe to intercalation into DNA leading to inhibition of synthesis of macromolecules, generation of reactive oxygen species, DNA binding and DNA cross-linking and DNA damage by inhibition of topoisomerase II, and eventually induce apoptosis or/and cell cycle arrest [7, 9, 10]. Due to drug resistance and the severe side effects, single-agent chemotherapy is no longer appropriate for treating human tumors. Recently, combination (rather than single-agent) chemotherapy has been found to be a superior treatment strategy [1, 11-13]. Hence, much effort has been made to identify chemo-sensitizers, that is, agents that are able to augment efficiency of anticancer drugs and simultaneously overcome multi-drug resistance (MDR) and side effects [14-16].

Selenium (Se), as an essential mineral trace element, is of much importance to animals and humans [17]. Much evidences demonstrates that supplement of Se can effectively reduce cancer risk [18-20]. Additionally, recently studies indicated that Se has a potential to increase the efficiency of cancer therapy where in combination with other anticancer drugs [21, 22]. For instance, Li *et al* reported that selenium can synergizes MCF-7 human breast cancer cells to DOX-induced apoptosis through modulation of phosphorylated AKT and its downstream substrates [21]. However, the apoptotic mechanism induced by Se-compounds remains elusive, especially this synergistic effect of Se combination treatment.

Selenocystine (SeC) is a naturally available selenoamino acid, which exhibits broad-spectrum anti-proliferative activity against several human tumor cells through oxidative stress-mediated apoptosis [23]. Despite this potency, SeC showed less toxic to HK-2 human normal cells, indicating the splendid selectivity of SeC and predicting the potential application in cancer chemoprevention [23, 24]. Moreover, we just reported that SeC can synergize auranofin-induced apoptosis in MCF-7 human breast cancer cells through triggering ROS-mediated DNA damage by triggering thioredoxin reductase (TrxR) [25]. In this study, we evaluated the ability of SeC to synergize the inhibitory action of DOX in HCC cells, and mechanistic investigation elucidated that SeC as a potential chemo-sensitizer can dramatically enhances DOX-induced cell killing against HCC cells *in vitro* and *in vivo* by triggering ROS-mediated DNA damage. Our study indicates that the strategy to use SeC and DOX in combination could be a highly efficient way to achieve anticancer synergism.

## RESULTS

### SeC enhances DOX-induced HepG2 cell killing by enhancing intracellular DOX accumulation

**Figure 1:**
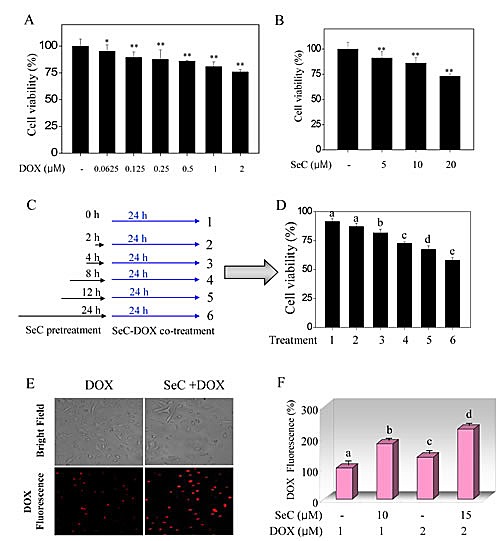
SeC enhances DOX-induced growth inhibition against HepG2 cells through enhanced intracellular uptake of DOX Cytotoxic effect of DOX (A) and SeC (B) on HepG2 cells. HepG2 cells (2×10^3^ cells/well) were seeded in 96-well plate and pre-incubated for 24 h. After incubation, cells were treated with indicated concentration of DOX for 24 h or SeC for 48 h. Cell viability was detected by MTT assay. (C, D) Combined treatment enhanced growth inhibition against HepG2 cells induced by SeC and DOX. The cells were pre-treated with 10 μM SeC (0, 2, 4, 8, 12 and 24 h) and co-incubated with 100 nM DOX for 24 h. Cell viability was determined by MTT assay. Intracellular uptake of DOX was detected by fluorescence microscope (C) and fluorescence micro-plate reader (D). Cells were pre-treated with SeC 24 h and/or DOX for 24 h and assayed by fluorescence microscope (Magnification, 100×) and fluorescence micro-plate reader as described in section of methods. Bars with different characters are statistically different at P<0.05 level.

Screening experiments were performed to ascertain the time and dose of SeC with DOX for the combined treatment in HepG2 cells. Treatment of HepG2 cells with SeC for 48 h or with DOX for 24 h alone inhibited the cell growth in dose-dependent manner (Fig. 1A and B). Interestingly, combined treatments of the cells with SeC and DOX significantly enhanced cell growth inhibition (Fig. 1C, D and Fig. S1A). For instance, treatment of HepG2 cells with 10 μM SeC for 48 h or with 100 nM DOX for 24 h reduced cell viability by 13.8 % and 7.2 %, respectively. Nevertheless, combination of 10 μM SeC (pretreatment for 24 h) with 100 nM DOX (co-treatment for another 24 h) significantly decreased the cell viability by 38.8% (Fig. S1A), implying that SeC effectively enhanced DOX-induced growth inhibition against HepG2 cells. Cell morphological change further confirmed the growth suppressive effect on HepG2 cells (Fig. S1B). Additionally, the interaction mode between SeC and DOX was examined by interaction index (γ) by isobologram analysis. Preliminary studies found that the IC_50_ value of SeC and DOX alone is 46.4 μM and 2.9 μM, respectively. Moreover, treatment of cells with 20 μM SeC in combination with 0.5 μM DOX displayed 50% cell killing. Therefore, the interaction index was γ=0.6<1, meaning that the combined effects between SeC and DOX was strongly synergistic.

DOX is an auto-fluorescent agent, allowing the visualization of its intracellular presence by fluorescence microscopy. To investigate the synergetic effect of SeC and DOX, intracellular accumulation of DOX was examined by detecting the intracellular fluorescence of DOX in HepG2 cells after treatment with SeC or /and DOX. As expected, HepG2 cells after treatment with 100 nM DOX alone showed weak red fluorescence in the nuclei (Fig. 1E). However, the intracellular fluorescence of DOX in HepG2 cells after pretreatment with 10 μM SeC was remarkable higher than that of DOX treatment alone (Fig. 1E and F). The enhanced fluorescence intensity of DOX further confirmed this conclusion that, SeC pretreatment enhanced DOX uptake in HepG2 cells (Fig. 1 F). Taken together, these results indicate that SeC enhances DOX-induced cell killing ability through enhancing its cellular uptake.

### SeC and DOX induce cell apoptosis and cell cycle arrest

**Figure 2:**
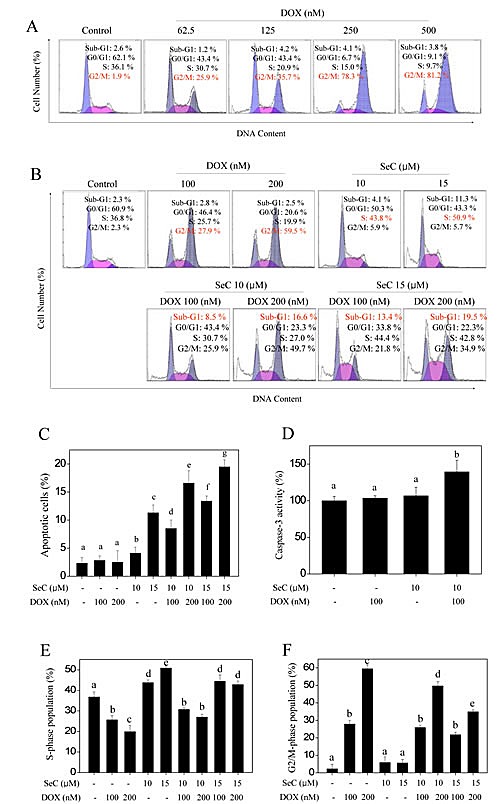
Apoptosis and cell cycle arrest induced by SeC or/and DOX A. DOX triggers G2/M cell cycle arrest in HepG2 cells in dose-dependent manner. HepG2 cell were pre-treated with 10 μM SeC for 24 h and co-treated with 100 nM DOX for 24 h. B. Combined treatment with SeC and DOX triggers apoptosis and cycle arrest. Cells after treatment were collected and stained with PI solution after fixation by 70% ethanol. Then cells was analyzed by flow cytometric analysis. Apoptotic cells with hypodiploid DNA content were measured by quantifying the sub-G1 peak. Statistical analysis of apoptotic cell (C) and cell cycle population (E and F). D. Caspase-3 activity. Cell after treatment were lysed and the cell proteins was used to determine caspase-3 activity using specific fluorescence substrates.

To determine the cause of combined treatment-induced cell death is apoptosis or/and cell cycle arrest, PI staining detected by flow cytometry was employed to evaluate combined treatment-induced cell death. HepG2 cells treated with indicated concentration of DOX initiated an apparent G2/M-phase cell cycle arrest with a dose-dependent manner, no obvious cell apoptosis was observed (Fig. 2A). Exposure of HepG2 cells to indicated concentration of SeC caused a marked cell apoptosis and S-phase cell cycle arrest (Fig. 2B). However, combined treatment of HepG2 cells with SeC (10 and 15 μM) and DOX (100 and 200 nM) resulted in a enhanced cell apoptosis compared with that of SeC and DOX alone, as reflected by the increase in sub-G1 peaks (Fig. 2B, C). Furthermore, DOX-induced G2/M-phase cell cycle arrest was blocked at S-phase by SeC (Fig. 2E, F). Enhanced cell apoptosis induced by combined treatment was confirmed by caspase-3 activity using substrate Ac-DEVD-AMC. As shown in Fig. 2D, pretreatment of HepG2 cells with SeC significantly enhanced the DOX-induced activation of caspase-3 from 103.6% (DOX), 106.8% (SeC) to 139.5% (combination). Taken together, these results suggest that SeC can act as an enhancer to sensitizes DOX-induced HepG2 cell killing by induction of apoptosis and cell cycle arrest.

### ERK and AKT pathways contribute to the combined treatment-induced growth inhibition against HepG2 cells

**Figure 3:**
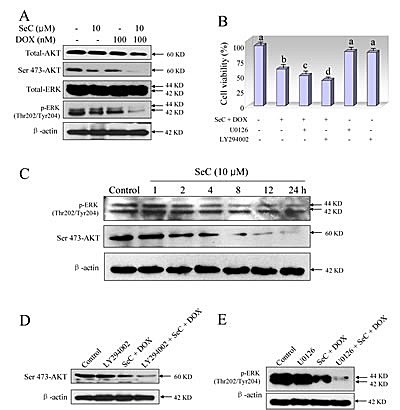
Contribution of ERK and AKT pathways to the combined treatment-induced growth inhibition against HepG2 cells A. SeC synergizes DOX-induced inactivation of ERK and AKT. Protection expression was detected by western blotting method. Briefly, HepG2 cell were pre-treated with 10 μM SeC for 24 h and co-treated with 100 nM DOX for 24 h. Then cells were lysed and total protein were separated by SDS-PAGE and immunoblotted with specific primary antibodies. Equal loading was affirmed by stripping immunoblots and reprobing for β-actin. B. Inhibitors of ERK (U0126) and AKT (LY294002) enhance combined treatment-induced growth inhibition against HepG2 cells. Cells were pretreated with 10 μM U0126 or 10 μM LY294002 for 1 h before combined treatment with SeC and DOX. Cell viability was detected by MTT assay. C. Time-dependent effects of SeC on expression of p-AKT and p-ERK in HepG2 cells. D. Inhibitors of AKT (LY294002) enhance combined treatment-induced inactivation of AKT. E. Inhibitors of ERK (U0126) enhance combined treatment-induced inactivation of ERK.

Accumulated evidences indicate that HCC cell activation by different factors is known to increase MEK/ERK and PI3K/AKT signaling [26]. To examine whether MEK/ERK and PI3K/AKT signaling involved in combined treatment-induced HCC cell growth inhibition, western blotting method was employed to detect ERK and AKT expression *in vitro* using phosphorylated antibodies. As shown in Fig. 3A, treatment of HepG2 cells with SeC and DOX alone both slightly inhibited the phosphorylation of ERK (Thr 202/Tyr 204) and AKT (Ser 473). However, pretreatment of HepG2 cells with SeC significantly enhanced DOX-induced inactivation of ERK and AKT (Fig. 3A). To further evaluate the role of SeC, a time course effect of SeC on ERK and AKT expression was assayed. As expected, exposure of HepG2 cells to 10 μM SeC for indicated time resulted in a notable down-regulation of ERK and AKT expression in time-dependent manner (Fig. 3C), indicating that SeC can act as an inhibitor of ERK and AKT to synergize DOX-induced HepG2 cell growth inhibition. Based on the importance of ERK and AKT, two inhibitors of ERK (U0126) and AKT (LY294002) were used to determine combined treatment-induced HepG2 cell killing. The MTT assay suggested that pretreatment with inhibitors only for 1 h obviously enhanced combined treatment-induced cell growth inhibition against HepG2 cells (Fig. 3B). Pretreatment of the cells with inhibitors of AKT (LY294002) and ERK (U0126) for 1 h effectively enhanced the combined treatment-induced dephosphorylation of ERK and AKT (Fig. 3 D and E). These results all indicated that combined treatment reduced HepG2 cell viability with ERK- and AKT-dependent manner. SeC as inhibitors of ERK and AKT signaling synergizes DOX-induced growth inhibition against HepG2 cells.

### SeC enhances DOX-induced DNA damage through ROS overproduction

**Figure 4:**
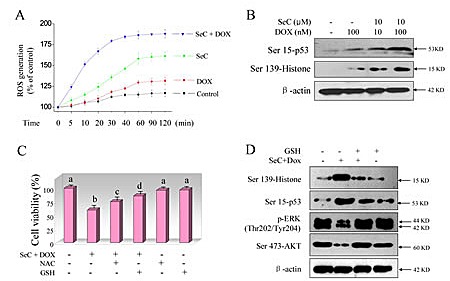
SeC synergizes DOX-induced DNA damage through ROS overproduction A. SeC enhances DOX-induced ROS accumulation. HepG2 cells (105 cells/well) were pretreated with 10 μM SeC for 1 h and co-incubated with 100 nM DOX for another 1 h, and then the time-course of ROS generation was measured by a fluorescence probe, DCFH-DA. B. SeC enhances DOX-induced phosphorylation of p53 and histone. HepG2 cell were pre-treated with 10 μM SeC for 24 h and co-treated with 100 nM DOX for 24 h. Then cells were lysed and the protein expression was examined by western blotting methods. C. Inhibition of ROS elevates the cell viability induced by combined treatment. Cells were pre-treated with 5 mM GSH or NAC for 2 h before combination treatment. Trypan blue staining was employed to quantify cell death. D. GSH supplement attenuates DOX-induced DNA damage and inactivation of ERK and AKT. Protein expression was detected by western blotting method.

DOX is reported to produce ROS as a part of its anticancer mechanisms [9]. Our previously proved that SeC can induced several human cancer cells apoptosis through ROS-mediated oxidative damage [23]. Hence, we asked whether SeC could enhance the ROS accumulation induced by DOX. As expected, SeC treatment alone caused apparent ROS overproduction in time-dependent manner as convinced by the enhanced green fluorescence intensity (Fig. S2). SeC pretreatment significantly enhanced DOX-induced ROS generation in time-dependent manner, and the ROS generation was observed as early as 5 min (Fig. 4A), indicating the significance of ROS as early event in regulating cell apoptosis or/and cell cycle arrest. Abundant intracellular ROS may cause DNA damage and activate down-streamed signaling pathway. Therefore, combined treatment-induced DNA damage was also detected using two DNA damage markers, phosphorylated p53 (Ser 15) and phosphorylated histone (Ser 139). As shown in Fig. 4B, treatment with SeC and DOX alone both triggered DNA damage, as convinced the up-regulated phosphorylated level of p53 and histone. However, combined treatment caused more severe DNA damage (Fig. 4B). Moreover, the combined treatment-induced DNA damage was further confirmed by DAPI staining. As shown in Fig. S3, pretreatment of HepG2 cells with SeC significantly enhanced DOX-induced chromatin condensation. Synergistic induction of DNA fragmentation by SeC and DOX was further confirmed by PI flow cytometric analysis, as evidenced by the increase of Sub-G1 peak (Fig. 2). We speculated the possibility that overproduced ROS induced by SeC transfered into nucleus to enhance DOX-mediated DNA damage. For further evaluation of ROS, two thiol-reducing antioxidant, glutathione (GSH) and N-acetyl-L-cysteine (NAC), were introduced to examine the role of intracellular ROS in combined treatment-induced cell death. As shown in Fig. 4C, pretreatment with 5 mM NAC or GSH for 2 h effectively prevented HepG2 cells from combined treatment-induced cell growth inhibition. For instance, combination treatment of SeC (10 μM) with DOX (100 nM) decreased the cell viability to 59.9%. However, pretreatment of cells with 5 mM NAC or GSH reversed the combined treatment-induced reduction of cell viability to 75.6% and 86.2%, respectively (Fig. 4C). In addition, GSH supplement effectively suppressed combined treatment-induced DNA damage and attenuated the inactivation of ERK and AKT (Fig. 4D), revealing the importance of ROS in DNA damage and regulation of ERK and AKT pathways.

### SeC amplifies the therapeutic effect of DOX in vivo

**Figure 5:**
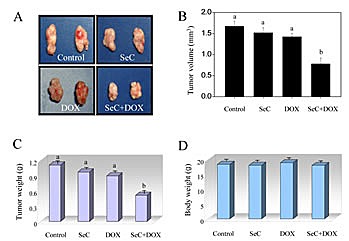
SeC enhances DOX-induced growth inhibition of tumor xenografts Combined treatment inhibits tumor volume (A, B) and tumor weight (C) of HepG2 human hepatoma carcinoma xenografts in nude mice, but not affect body weight (D) of mice.

**Figure 6:**
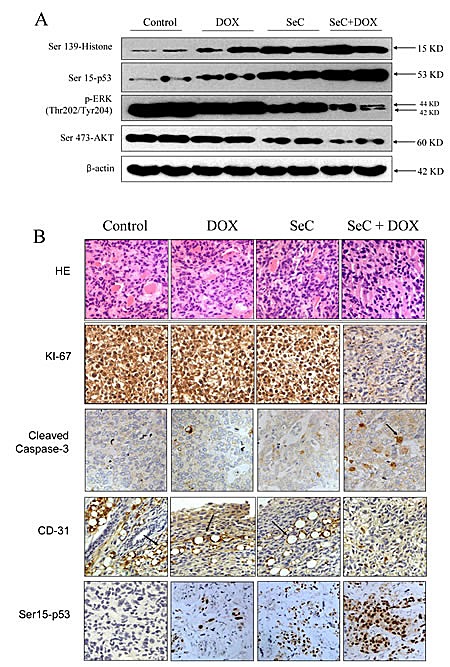
Combined treatment triggers DNA damage and inactivation of ERK and AKT in vivo A. Proteins expression detected by western blotting in vivo. B. Staining of tumor xenografts by HE and IHC method.

To evaluate the synergetic effect of SeC and DOX *in vivo*, immuno-deficient nude mice bearing HCC tumor xenografts were employed to investigate the therapeutic effect of SeC in combination with DOX. As expected, after 14 days' administration, treatment of SeC and DOX alone both slightly inhibited HCC tumor xenografts growth. However, HCC tumor xenografts growth in rude mice was more effectively inhibited by combined treatment with SeC and DOX *in vivo*. For instance, combined treatment with SeC and DOX significantly inhibited the tumor volume (Fig. 5A, B) and tumor weight (Fig. 5C), but not affected body weight of mice (Fig. 5D). The *in vivo* mechanistic studies revealed that combined treatment inhibited tumor xenografts by induction of AKT and ERK inactivation and triggering DNA damage (Fig. 6 A), which were consistent with the results *in vitro*. Furthermore, several cell markers using IHC methods further confirmed that combined treatment triggered cell apoptosis (cleaved caspase-3 staining), inhibited angiopoiesis (CD31 staining), activated p53 phosphorylation (Ser15-p53 staining) and inhibited tumor xenografts cell proliferation (Ki67 staining) (Fig. 6B). Statistic analysis of the protein expression levels by Leica QW550 software further confirmed this anti-tumor effect *in vivo* (Fig. S4). Taken together, these results all indicated that SeC synergizes the therapeutic effect of DOX *in vivo* by DNA damage-mediated p53 phosphorylation.

## DISCUSSION

In the present study, we validated for the first time that SeC synergizes DOX-mediated HepG2 cell killing *in vitro* and *in vivo* and the possible mechanisms. The results show that DOX inhibited the growth of HepG2 cells by induction of G2/M cell cycle arrest, and SeC induced growth inhibition against HepG2 cell through S-phase arrest and cell apoptosis. However, combined treatment with DOX and SeC significantly inhibited the growth of HepG2 cells by dual-cell cycle arrest and enhanced cell apoptosis. Inhibition of HepG2 cells induced by combined treatment was associated with increased ROS generation, DNA damage, and inactivation of ERK and AKT pathway.

DOX as one of the most effective chemotherapeutic agent was widely used for treating human various malignancies [6-8, 20]. Nevertheless, the side effects, especially the severe cardiotoxicity, badly limited its clinical usage. In the present study, we used SeC, a low cytotoxic agent, to synergize the therapeutic effect of DOX, and the result suggested that the combined treatment effectively induced the HepG2 cell killing. Meanwhile, the combined strategy reduces the effective dosage of DOX (only 100 nM), but enhance the pharmaceutical effect of DOX, which has the potential to minimize/reverse the side effects and multi-drug resistance (MDR) of DOX with promising application in clinic.

Induction of cell apoptosis or/and cell cycle arrest by chemo-preventive and chemotherapeutic agents in cancer cells is an effective strategy to halt tumor growth [27]. The tumor suppressor p53 is a cell cycle checkpoint protein that contributes to the preservation of genetic stability by mediating either cell cycle arrest or apoptosis in response to DNA damage [28, 29]. The major molecular sensors, including ATM, ATR, and DNA-PK, can be recruited in response to DNA damage, accompanied by the activation of p53 and downstream pathway [30-32]. Finally, activated p53 can cause cell cycle arrest or apoptosis to repair/eliminate the damaged cells [33-37]. In the present study, treatment with DOX and SeC alone both triggered the phosphorylation of p53 and histone, indicating that DOX and SeC both induced DNA damage. Combined treatment resulted in enhanced DNA damage, as convinced by increased expression of p53 and histone. Activation of p53 in response to DNA damage finally induced apoptosis and cell cycle arrest in HepG2 cells.

Recent studies found that the Ras/Raf/MAPK and PI3K/AKT/mTOR are two activated pathways in HCC cells [26, 32, 38-40]. The PI3K/AKT/mTOR signaling pathway plays a pivotal role in HCC and is activated in 30-50% of HCC cases [26]. Therefore, searching for effective inhibitors targeting these signaling pathway or their components represents a promising therapeutic strategy recently. In this study, we showed that the combined treatment with SeC and DOX notably decreased ERK and AKT phosphorylation in cancer cells. Furthermore, pretreatment of the cells with inhibitor of ERK and AKT significantly enhanced the combined treatment-induced cell killing, indicating that ERK and AKT pathway both contributed to combined treatment-induced growth inhibition against HepG2 cells. The results indicated that SeC as potential inhibitors of ERK and AKT showed promising application in treating human hepatocellular carcinoma.

Human tumor cell killing by chemotherapy agents was usually ascribed to the accumulation of intracellular ROS [41]. Overproduction of intracellular ROS may attack cellular membrane lipids, proteins, DNA and cause oxidative injury, and finally result in reduction of cell cycle arrest or/and cell apoptosis to repair or eliminate the damaged cells [42]. DOX is known to produce ROS as a part of its anticancer mechanisms [9]. Our previous studies found that SeC could induce several human cancer cell apoptosis by induction of ROS generation [23]. Consistent with expected results, we confirm that DOX and SeC treatment alone both caused significant ROS accumulation. Although ROS generation was greater in SeC-treated cells, combination of DOX with SeC further enhanced ROS overproduction.

In view of ROS production, we performed the western blotting method to assess DNA damage using specific phosphorylated antibody, Ser 15-p53 and Ser 139-histone, both DNA damage markers. The results showed that SeC and DOX pretreatment alone both slightly caused DNA damage in HepG2 cells, as convinced by the up-regulation of Ser 15-p53 and Ser 139-histone. Although DNA damage level was higher in SeC-treated cells, combination treatment with DOX and SeC resulted in severer DNA damage in HepG2 cells. However, blocking of ROS production with

ROS scavengers, GSH and NAC, both thiol-inducing antioxidants, effectively prevented HepG2 cells from combined treatment-mediated cell killing. Moreover, elimination of ROS by GSH also significantly attenuated phosphorylation of histone and inactivation of ERK and AKT, indicating that ROS act as a early mediator in regulating DNA damage and ERK and AKT pathway. These results provided a possibility that, SeC inside the cells may oxidize intracellular thiol-containing antioxidant agents. Addition of GSH and NAC not only eliminated ROS, but also replenished intracellular stores of endogenous thiol-containing antioxidants. Our previous studies have already confirmed the conclusion that, ROS as upstream mediator can regulation the phosphorylation of ERK and AKT. Meanwhile, addition of GSH effectively inhibited ROS generation and suppressed the dephosphorylation of ERK and AKT [34]. Taken together, our results suggest that ROS overproduction lead to the induction of DNA damage, and inactivation of ERK and AKT, leading to the activation of caspase-3 to induce apoptosis and cell cycle arrest.

In summary, we combined DOX with SeC to enhance HepG2 cell killing by induction of apoptosis and cell cycle arrest through ROS-mediated DNA damage. We also demonstrated the synergistic effect of DOX/SeC combination on suppression of tumor growth *in vivo* using a xenograft tumor model. Our results suggest that combining low dose of DOX with suboptimal dose of SeC can serve as a potential combination therapy for treatment of human hepatocellular carcinoma with the potential to minimize/eliminate the side effects associated with high doses of DOX.

## MATERIALS AND METHODS

### Reagents

Selenocystine, doxorubicin, propidium iodide [1], 2',7'-dichlorofluorescein diacetate (DCF-DA), 3-[4,5-dimethyl-thiazol-2-yl]-2,5-diphenyltetrazolium bromide (MTT), bicinchoninic acid (BCA) kit for protein determination were purchased from Sigma. Dulbecco's modified Eagle's medium (DMEM), fetal bovine serum (FBS) and the antibiotic mixture (penicillin-streptomycin) were purchased from Invitrogen (Carlsbad, CA). Caspase-3 substrate (Ac-DEVD-AMC) were purchased from

Calbiochem. U0126 and LY294002 were obtained from Calbiochem (San Diego, CA). All of the antibodies used in this study were purchased from Cell Signaling Technology (Beverly, MA). All of the solvents used were of high-performance liquid

chromatography (HPLC) grade. The water used for all experiments was supplied by Milli-Q water purification system from Millipore.

### Cell culture

HepG2 hepatocellular carcinoma cell line was obtained from American Type Culture Collection (ATCC, Manassas, VA) and maintained in DMEM medium supplemented with fetal bovine serum (10%), penicillin (100 units/ml) and streptomycin (50 units/ml) at 37°C in a humidified incubator with 5% CO_2_ atmosphere.

### Cell viability

Briefly, HepG2 cells (2×10^3^ cells/well) seeded 96-well micro-plates were pre-treated with 10 μM SeC for 24 h and co-incubated with 100 nM DOX for another 24 h. After incubation, cell viability was detected by MTT assay as previously reported [24].

### Intracellular uptake of DOX

The intracellular uptake of DOX was visualized by fluorescence microscopy and quantified by fluorescence micro-plate reader. Briefly, HepG2 cells (1×10^5^) were seeded in 2-cm culture dish and pre-incubated for 24 h. After incubation, cells were pre-treated with 10 μM SeC for 24 h in the presence or absence of 1 μM DOX for another 24 h. After treatment, the cells were washed with PBS for 3 times and the intracellular uptake of DOX was detected by fluorescence microscopy (Magnification, 100×). In addition, The intracellular uptake of DOX was quantified by fluorescence micro-plate reader. Briefly, cells were seeded in 96-well micro-plate (2×10^3^ cells/well) and treated with SeC (10 μM) or/and DOX (1 μM). After treatment, cells were washed with PBS for 3 times and the intracellular uptake of DOX was quantified by fluorescence micro-plate reader with the excitation wavelength and emission wavelength at 485 nm and 585 nm, respectively. The data was expressed as % of DOX-treated group (100%).

### Cell apoptosis and cell cycle distribution

Briefly, cells after treatment with SeC or/and DOX were harvested by centrifugation and washed with PBS. Cells were stained with PI after fixation with 70% ethanol at −20°C overnight. Then the cell apoptosis and the cell cycle distribution was analyzed by flow cytometric analysis as previously described [24].

### Determination of caspase-3 activity

Total protein in HepG2 cells exposed to SeC or/and DOX was extracted and the protein concentration was quantified by BCA kit. Then the activity of caspase-3 was detected as previously described [24].

### Intracellular ROS accumulation

Intracellular ROS accumulation was detected by DCF fluorescence assay. Briefly, HepG2 cells (1×10^6^ cells/ml) in PBS were incubated with 10 μM DCFH-DA at 37°c for 45 min. Then the cell were washed and the labelled cells were placed in 96-well plate (100 μl/well) and incubated with 10 μM SeC for 1 h. After then, the cells were co-incubated with 100 nM DOX for another 1 h. The intracellular ROS level was determined as previously described [23].

### Western blot analysis

The total cellular proteins in HepG2 cells exposed to SeC and/or DOX were extracted and quantified using BCA kit according to the manufacturer's protocols. Then the proteins expression in HepG2 cells after treatment were investigated by western blotting method as previously described [25].

### In vivo study

Male nude mice were used to evaluate the *in vivo* therapeutic effect of SeC or/and DOX. Briefly, about 1×10^6^ HepG2 cells in 100 μL serum-free medium were subcutaneously injected into the right oxter of mice. When average tumor volume reached about 50 mm^3^ after 1 week, mice were randomly divided into four groups (8 mice/group): Group 1 for PBS as control; Group 2 for 5 mg/kg SeC; Group 3 for 2 mg/kg DOX; Group 4 for 5 mg/kg SeC + 2 mg/kg DOX. Drugs were injected every other day, caudal vein, from the first day until the fourteenth day (7 times). At the termination of the experiments, tumors were harvested, photographed and weighed. Tumor dimensions were measured with calipers and the volume was calculated using the formula: volume = l×w^2^/2, with l being the maximal length and w being the width. A portion of the tumors from control and treated animals was used for preparation of tumor lysate used in further analysis. Another portion of tumors were removed, fixed in 10% buffered formalin, embedded with paraffin and sectioned. The 4-μM sections were stained with hematoxylin and eosin staining (HE) for histological observation. Protein expression in sections was examined by immunohistochemical (IHC) methods. All animal experiments were approved by the Animal Experimentation Ethics Committee.

### Statistical analysis

Experiments were carried out at least in triplicate and repeated three times. All data were expressed as mean ± S.D. Statistical analysis was performed using SPSS statistical package (SPSS 13.0 for Windows; SPSS, Inc. Chicago, IL). The difference between two groups was analyzed by two-tailed Student's t-test. The difference between three or more groups was analyzed by one-way analysis of variance multiple comparisons. Differences with *P*<0.05 (*) or *P*<0.01 (**) was considered statistically significant. Bars with different characters are statistically different at *P*<0.05 level. The interaction mode between SeC and Dox was analyzed by the interaction index which is based on “isobologram analysis” [34, 43]. The index, denoted by γ, is defined by the isobolar relation: a/A + b/B = γ, where A and B are the doses of SeC and DOX used, which give 50% cell killing, and a and b are the combination doses that produce the same activity. The quantities in equation are obtained from the dose response curves of drugs A, B, and the combined treatments. If γ=1, the interaction is additive; if γ<1, it is synergistic; if γ>1, it is antagonistic.

## SUPPLEMENTARY FIGURES


