# Supplementary material for: Strategy to enhance the therapeutic effect of doxorubicin in human hepatocellular carcinoma by selenocystine, a synergistic agent that regulates the ROS-mediated signaling
Source: Oncotarget. 2014 Mar 25;5(9):2853–63. doi: 10.18632/oncotarget.1854 (PMC4058050; doi:10.18632/oncotarget.1854)
Supplement: Supplementary file 1 [file oncotarget-05-2853-s001.pdf]

Strategy to enhance the therapeutic effect of doxorubicin in human hepatocellular carcinoma by selenocystine, a synergistic agent that regulates the ROS-mediated signaling

Cundong Fan[1], Wenjie Zheng[*,1], Xiaoyan Fu[1], Xiaoling Li[1], Yum-Shing Wong [2], Tianfeng Chen[*,1]

[1] Department of Chemistry, Jinan University, Guangzhou 510632, China

[2] School of Life Sciences and State Key Laboratory of Agrobiotechnology, The Chinese University of Hong Kong, Hong Kong S.A.R., China

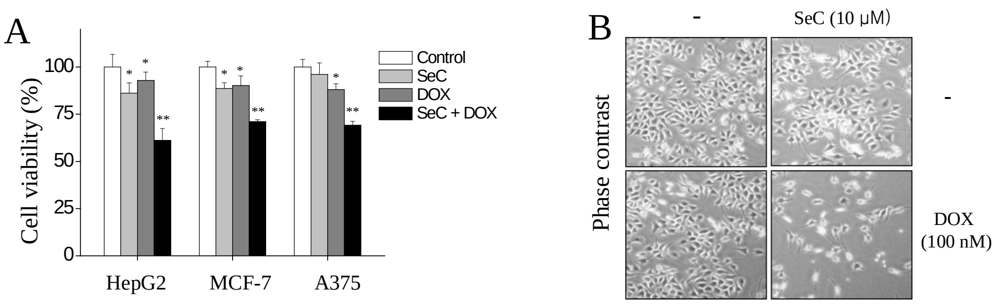

Figure S1. Cytotoxicity of SeC and DOX towards human cancer cells. A. Combined treatment-induced growth inhibition against human cancer cells. The cells exposed to 8 μM SeC for 24 h were co-incubated with 100 nM DOX for 24 h. Cell viability was determined by MTT assay. **B**. Changes of cell morphology. HepG2 cells after treatment with SeC (10 μM) or/and DOX 100 nM were observed by phase contrast microscope (Magnification, 200×). All data here are expressed as means ± SD of triplicates. The image shown here is representative of three independent experiments with similar results.

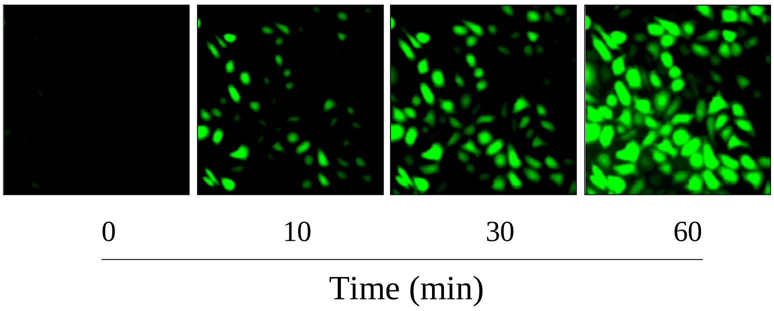

Figure S2. SeC induces time-dependent intracellular ROS accumulation. HepG2 cells were treated with 10 μM SeC for 0, 10, 30 and 60 min and then imaged by fluorescence microscope (magnification, 200×). The image shown here is representative of three independent experiments with similar results.

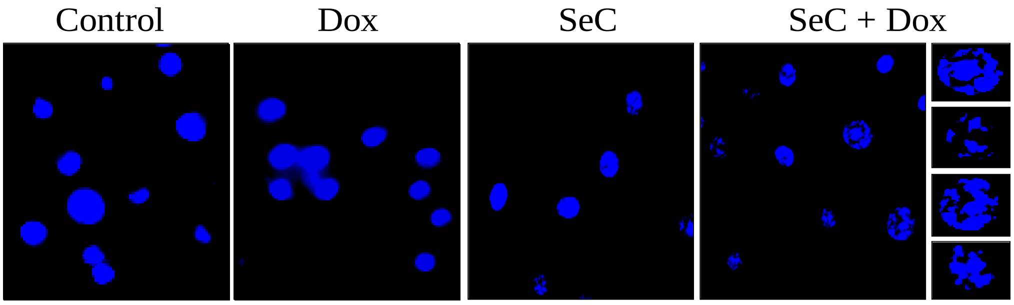

Figure S3. SeC enhances Dox-induced DNA damage. HepG2 cells cultured in chamber slides were pretreated with 10 μM SeC for 24 h and co-treated with Dox for 24 h. Then cells were fixed with 3.7% formaldehyde for 10 min and incubated with 1 μg/ml of DAPI for 15 min at 37℃. The cells were then washed with PBS and examined under a fluorescence microscope (magnification, 200×). The image shown here is representative of three independent experiments with similar results.

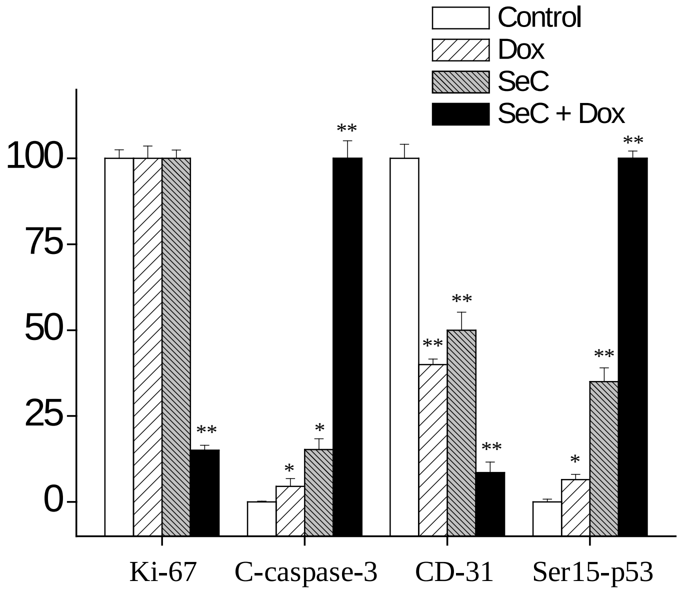

Figure S4. Statistic analysis of proteins expression *in vivo*. The protein expression was examined by IHC methods and analysed by Leica QW550 software. All data here are expressed as means ± SD of triplicates
